# Assessing attitudes towards elements of the overdose response hotlines/applications (ORHAs)

**DOI:** 10.1186/s12954-026-01411-3

**Published:** 2026-02-07

**Authors:** Avnit Dhanoa, Dylan Viste, Boogyung Seo, Nathan Rider, S. Monty Ghosh

**Affiliations:** 1 Department of Medicine, Faculty of Medicine and Dentistry, University of Alberta, Edmonton, AB Canada; 2 Department of Medicine, Cumming School of Medicine, University of Calgary, Calgary, AB Canada

**Keywords:** Overdose technologies, Harm reduction, Attitudes

## Abstract

**Introduction:**

In response to the overdose epidemic, novel strategies including Overdose Response Hotlines and Applications (ORHAs) have been introduced to help mitigate the crisis. These technologies enable individuals with a phone to access harm reduction support via smartphones and applications. Such supports include overdose monitoring, access to social services, mental health referrals, and more. This study analyzed data from the Canadian National Questionnaire on Overdose Monitoring (CNQOM), a large bilingual national survey, to evaluate the perspectives of people who use unregulated substances currently (PWUS-C), people who used unregulated substances previously (PWUS-P), and addiction service provider (ASP) on the importance of specific ORHA features.

**Methods:**

One component of the CNQOM pertained to the importance of specific ORHA service elements. Examined categories included accessibility and technological features, overdose response functionality, data privacy and philosophies of care, additional support services, and substance usage. Each group responded to 33 questions on a 5-point Likert scale, and the data was analyzed using descriptive statistics involving percentages and ordinal logistical regression analysis.

**Results:**

The study involved 971 participants: 840 PWUS-C, 298 PWUS-P, and 169 ASP. The majority of respondents from the key groups considered all ORHA elements important. Generally, the groups ranked the elements in a similar order of importance, with only minor variations. The highest-ranked elements in each element category with regards to importance were: 24/7 availability (84% of PWUS-C, 88% of PWUS-P, and 90% of ASP), the ability of EMS to resuscitate individuals during an overdose (81% of PWUS-C, 83% of PWUS-P, 85% ASP), non-judgmental support (87% of PWUS-C, 87% of PWUS-P, and 91% of ASP), access to mental health support (82% of PWUS-C, 84% of PWUS-P, and 90% of ASP), and feeling safer when using substances (80% of PWUS-C, 81% of PWUS-P, and 88% of ASP).

**Conclusion:**

This paper highlights the importance multiple groups place on various elements of ORHAs, reflecting critical elements that should be considered when standardizing these virtual harm reduction technologies. The results of this study provide insight into opportunities to enhance virtual platforms, making them more responsive, accessible, and trusted as harm reduction resources.

## Introduction

From January to March 2024, there were 1906 fatal opioid-related overdoses in Canada, with an average of 21 deaths per day [1]. In response to the overdose epidemic, harm reduction measures such as Safe Consumption Sites (SCS) have been implemented to provide people who use substances (PWUS) a safe space to use substances, obtain sterile supplies, receive drug checking, and get access to other support services [2]. SCS can also help reduce the spread of infectious diseases, like HIV, while reducing the risk of overdoses [3]. Although SCS have helped combat the overdose crisis, studies have shown there are certain drawbacks. For instance, business owners have been wary of allowing SCS to operate near their premises and inhalation is one popular method of opioid and methamphetamine usage, but many SCS do not permit this route of consumption due to a lack of appropriate ventilation support [4, 5]. In the past 5 years, novel strategies such as Overdose Response Technologies (ORTs) have been implemented to help address the limitations of in-person harm reduction support [6]. These virtual harm reduction methods are based on the idea of “drug spotting”, which can provide monitoring for individuals who use alone, prefer inhalation methods, or are located in areas where there is a lack of SCS [7].

Overdose Response Technologies (ORT) are a broad category of interventions including fixed location devices, wearable technology, and lastly Overdose Response Hotlines and Applications (ORHAs). The latter category includes smartphone applications and hotline services, allowing anyone with a phone and/or internet access to access harm reduction support [6]. This support includes but is not limited to overdose monitoring. ORHAs can also provide social services referrals, mental health support, harm reduction education, recovery-oriented addiction supports, and more [8, 9]. The National Overdose Response Service (NORS) is an example of an ORHA, which connects PWUS with peer operators (individuals with lived experience of substance use) who monitor the caller for signs of overdose [10]. If the caller becomes unresponsive at any point, EMS is dispatched to the individual's location. Alternatively, a community-based responder, such as a friend or family member, can be contacted in accordance with the caller’s pre-agreed emergency response plan [10]. Other overdose response hotlines include Never Use Alone (an American hotline) and SafeSpot (another American hotline) [11]. Overdose response applications include the Digital Overdose Response Service (DORS) and the LifeGuard app. These apps start an emergency alarm if the client does not check in at regular time intervals, which can be followed by an emergency location-based response if the client does not disable the alarm [6].

Previous literature has explored how individuals perceive ORHAs [8, 12, 13]. While expressing concerns about EMS response times, PWUS have described feelings of optimism towards virtual overdose monitoring, as they can reduce mortality and increase confidentiality [8]. Additionally, qualitative research has shown that healthcare providers felt that ORHA bridges the gap between logistical barriers (e.g. long wait times, ventilation) and SCS [8]. Furthermore, the perspectives of women and gender-diverse individuals have been explored and ORHAs have been described as providing gender-based safety in comparison to physical sites [13].

While there has been research on how various individuals perceive ORHAs, more quantitative research needs to be done to explore how different groups view the service elements. Given the wide range of practical, operational, and implementation approaches to ORHAs, understanding the perspectives of key groups regarding specific service elements of interest could help inform quality improvement activities and funding decisions. It would also help elucidate the optimal ORHA service components and functionality. Incorporating multiple perspectives, specifically from individuals with lived or living experience of substance use, as well as service providers that support those who use substances can help guide improvements to tools designed to aid in harm reduction and substance use recovery [14]. As users of such technologies, PWUS can provide feedback about services and treatments [15]. In particular, considering the perspectives of PWUS can aid researchers in developing technology that uses clear and appropriate language and is reflective of their needs, while highlighting weaknesses in the tools [14]. Perspectives of service providers is also important, as they influence treatment decisions and healthcare outcomes [16]. Additionally, there can also be diverging perspectives between PWUS and professionals in the field, with both viewpoints being valuable in advancing harm reduction tools and fitting service offerings to the best evidence [16].

The aim of this paper is to explore data from a large bilingual national survey, the Canadian National Questionnaire on Overdose Monitoring [17], to assess perspectives of people who use substances currently (PWUS-C), people who used substances previously (PWUS-P), and addiction and harm reduction workers (ASP) on the level of importance of specific aspects of ORHAs such as, but not limited to, accessibility and technological logistics, overdose responses, data privacy and philosophies of care, additional support services, and substance usage.

## Methods

The study was approved by the University of Calgary’s Conjoint Health Research Ethics Board (CHREB; REB #21-1646). Study reporting was compliant with the STROBE Checklist for cross-sectional studies and the Checklist for Reporting Results of Internet E-Surveys (CHERRIES) [18, 19].

### Study design

The data in this paper was collected as a component of the Canadian National Questionnaire on Overdose Monitoring (CNQOM), a large-scale bilingual seven-component digital survey completed in 2023 [17]. One component of the CNQOM asked questions pertaining to the importance of specific ORHA elements, including accessibility and technological features, logistics, overdose responses, data privacy, addiction support services, and substance usage. Elements in this section of the survey were custom-designed to evaluate characteristics of ORHAs deemed to be important in the qualitative literature [8], from expert consultation (e.g. addiction medicine practitioners, first responders), and from involvement of harm reduction workers and PWUS. Two trap questions were included to assess the attention of the respondents. An iterative feedback approach was used to ensure comprehensiveness of the assessed ORHA elements. To ensure the use of non-stigmatizing language, three rounds of pilot testing were conducted by the principal investigator (MG) and the study team, which included a resident physician (NR), post-doctoral fellow (TM), research assistants (DV, WR, BS), healthcare professionals, and harm reduction workers. Furthermore, input from key partners was received to ensure face validity of each question. Experts from the field of harm reduction and addiction medicine also ensured content and construct validity. The survey was administered using the Qualtrics XM platform. Additionally, enabling IP address collection, duplicate response blocking, and creating multiple survey versions allowed for easier detection and mitigation of potentially fraudulent responses. Additional details about the survey’s data collection methods are further discussed in the CNQOM methods paper [17].

### Participants

A total of 971 participants from the CNQOM were included in this study (see Table 1 for participant demographics). Participant breakdown included 268 individuals who currently use unregulated substances (*Mage* = 37.02* SD* = 11.50), 525 individuals who previously used unregulated substances (*Mage* = 39.10* SD* = 13.54), and 178 addiction and harm reduction workers (*Mage* = 36.63* SD* = 11.44). Participants were recruited through purposive and random sampling from July 2022 to May 2023. The study team compiled a list of potential partner organizations that would have contacts for the groups and a standardized email was sent to each of these organizations. Organizations that responded to the initial email were sent additional information about the study. The study team provided distribution materials to partner organizations in the form of emails and posters that included a link to the survey and QR codes. All participants resided in Canada, were 18 years of age or older, able to access the online survey, and were able to communicate in English or French (Canada has two national languages). Participants had come from three different interested parties (PWUS-C, PWUS-P, and ASP). A $15.00 CAD honorarium was given to those with lived or living experience of substance use, who completed the survey using links from partner organizations through e-transfer or gift card. Participation was voluntary and electronic consent involved individuals reading over a digital consent form and selecting “I agree to participate” or “I DO NOT agree to participate”. If they did not consent, the survey ended. The survey had an estimated completion time of 15–25 min. Responses to eligible questions were mandatory and respondents did not have the option to go back to change responses.

**Table 1:** Demographic information of the participants, (where * represents multiple identified ethnicities)

	Total	PWUS-C	PWUS-P	ASP
Total	971	268 (27.6%)	525 (54.1%)	178 (18.3%)
Age				
Minimum	18	18	18	19
Maximum	82	79	82	78
Mean	39.10	37.02	41.01	36.63
Standard deviation	13.54	11.50	14.82	11.44
Gender				
Man	400 (41.2%)	124	239	37
Woman	515 (53.0%)	119	260	136
Gender-diverse	53 (5.5%)	24	25	4
Transgender man	14 (1.4%)	7	6	1
Transgender woman	5 (0.5%)	5	0	0
Non-binary	31 (3.2%)	10	18	3
Gender queer	1 (0.1%)	0	1	0
Gender fluid	1 (0.1%)	1	0	0
Two-spirit	1 (0.1%)	1	0	0
No gender	3 (0.3%)	1	1	1
Ethnicity				
White	715 (73.6%)	186	401	128
Indigenous	136 (14.0%)	48	74	14
Other ethnicities	108 (11.1%)	30	45	33
Multiple ethnicities	39 (4.0%)	12	24	3
Housing				
I live in a house that I own	383 (39.4%)	80	209	94
Renting apartment or house	427 (44.0%)	115	248	64
Living with a friend or family member	98 (10.1%)	34	45	19
Living in supportive housing	25 (2.6%)	13	11	1
Living in a shelter facility	15 (1.5%)	10	5	0
Currently unhoused	20 (2.1%)	14	6	0
Province				
Ontario	324 (33.4%)	98	173	53
Alberta	212 (21.8%)	67	95	50
British Columbia	110 (11.3%)	29	65	16
Québec	84 (8.7%)	22	50	12
Saskatchewan	80 (8.2%)	19	44	17
Manitoba	48 (4.9%)	10	27	11
New Brunswick	35 (3.6%)	6	21	8
Nova Scotia	41 (4.2%)	9	26	6
Newfoundland and Labrador	18 (1.9%)	3	11	4
Prince Edward Island	8 (0.8%)	2	5	1
Yukon	4 (0.4%)	0	4	0
Northwest Territories	3 (0.3%)	0	3	0
Nunavut	3 (0.3%)	2	1	0
Community size				
Large urban (> 100,000 people)	532 (54.8%)	158	280	94
Medium urban (30,000–99,999 people)	184 (18.9%)	51	99	34
Small urban (1000–29,999 people)	149 (15.3%)	33	82	34
Rural (< 1000 people)	76 (7.8%)	11	52	13

### Data analysis

Respondents answered 33 individual questions on a 5-point Likert scale, where “3” represented *neutral*, “1” represented *not very important* and “5” represented *very important*. Removal of identifying information (e.g. participant emails) was part of the data cleaning process. Missing responses underwent casewise deletion. Responses were also removed from analysis if they did not meet the inclusion criteria, had < 5% survey progress, had an IP address outside of Canada, completed the survey in less than 500 s, were flagged by Qualtrics as duplicate responses, or failed one or both of the trap questions. Ordinal logistical regression was performed to compare perspectives between the groups. We acknowledge that there are many ways to compute and interpret importance from ranked-order data, and that different analytic techniques may yield different ‘importance orderings’ and influence conclusions about the degree of importance of individual items. To ground our choice, we aimed to determine whether each element was important in an absolute sense, not to prioritize elements against one another. Likert scales are more appropriate for capturing absolute judgments compared to ranking procedures [20].

## Results

When asking interested parties (PWUS-C, PWUS-P, and ASP) to rank different ORHA elements, a majority of respondents deemed all aspects as important. Generally, respondents ranked the importance for elements similarly within each category, with only minor variations. When comparing rankings between each group, ASP ranked most elements of significantly higher importance than PWUS and individuals who used substances in the past. Herein, we examine the importance of the various service elements by considering the proportion of respondents who ranked each one “important” or “very important”. An ordinal logistical regression was also performed to assess the differences between group perceptions (Table 2).

**Table 2:** Ordinal logistic regression for predicting group’s perception towards service elements. The first group listed represents the numerator of odds ratio and the second group represents the denominator

		Odds ratio (95% Confidence interval)
Accessibility and technological logistics		
Availability of 24/7 service	ASP versus PWUS-C	1.58 (1.01–2.46)
	ASP versus PWUS-P	1.37 (0.91–2.06)
	PWUS-C versus PWUS-P	0.87 (0.63–1.20)
Service being free of charge	ASP versus PWUS-C	1.85 (1.18–2.91)
	ASP versus PWUS-P	1.82 (1.20–2.75)
	PWUS-C versus PWUS-P	0.98 (0.72–1.34)
Ability to use the service without a smartphone	ASP versus PWUS-C	1.82 (1.22–2.71)
	ASP versus PWUS-P	1.62 (1.13–2.34)
	PWUS-C versus PWUS-P	0.89 (0.67–1.20)
Having a reliable internet or phone connection	ASP versus PWUS-C	1.21 (0.84–1.76)
	ASP versus PWUS-P	1.16 (0.83–1.62)
	PWUS-C versus PWUS-P	0.95 (0.72–1.27)
Dropped calls or connectivity concerns	ASP versus PWUS-C	1.10 (0.75–1.62)
	ASP versus PWUS-P	1.27 (0.90–1.80)
	PWUS-C versus PWUS-P	1.16 (0.86–1.56)
Being able to text a peer operator for supervision or support	ASP versus PWUS-C	0.96 (0.66–1.40)
	ASP versus PWUS-P	1.02 (0.73–1.42)
	PWUS-C versus PWUS-P	1.06 (0.80–1.41)
Being connected to the same operator or set of operators each time (for telephone services)	ASP versus PWUS-C	0.80 (0.55–1.15)
	ASP versus PWUS-P	0.95 (0.69–1.32)
	PWUS-C versus PWUS-P	1.19 (0.90–1.58)
Being able to videocall or FaceTime a peer operator for supervision or support	ASP versus PWUS-C	1.24 (0.86–1.79)
	ASP versus PWUS-P	1.35 (0.97–1.87)
	PWUS-C versus PWUS-P	1.09 (0.82–1.44)
Overdose responses functionality		
Ability for EMS services to resuscitate you if you have a drug poisoning/ overdose	ASP versus PWUS-C	1.36 (0.91–2.03)
	ASP versus PWUS-P	1.29 (0.90–1.86)
	PWUS-C versus PWUS-P	0.95 (0.70–1.28)
An emergency button for apps like Brave, DORS, Connect by LifeGuard to get help immediately as opposed to waiting for the countdown to be completed	ASP versus PWUS-C	1.82 (1.22–2.71)
	ASP versus PWUS-P	1.53 (1.07–2.21)
	PWUS-C versus PWUS-P	0.84 (0.63–1.13)
Having an operator double check verbally to ensure you have not had a drug poisoning/overdose	ASP versus PWUS-C	1.66 (1.13–2.43)
	ASP versus PWUS-P	1.40 (0.99–1.99)
	PWUS-C versus PWUS-P	0.85 (0.64–1.13)
Call backs or check ins the day after a drug poisoning/overdose to ensure you or the client are okay	ASP versus PWUS-C	1.78 (1.21–2.63)
	ASP versus PWUS-P	1.70 (1.19–2.41)
	PWUS-C versus PWUS-P	0.95 (0.72–1.27)
Ability to use a community-based response where NORS and Brave can contact friends or family to support you in an event of a drug poisoning/overdose	ASP versus PWUS-C	1.53 (1.05–2.22)
	ASP versus PWUS-P	1.30 (0.93–1.82)
	PWUS-C versus PWUS-P	0.85 (0.64–1.13)
Automated countdowns (for DORS/Lifeguard) without need to talk to someone	ASP versus PWUS-C	1.49 (1.02–2.18)
	ASP versus PWUS-P	1.43 (1.02–2.02)
	PWUS-C versus PWUS-P	0.96 (0.72–1.28)
Data privacy and philosophies of care		
Non-judgmental support	ASP versus PWUS-C	1.72 (1.09–2.74)
Substance usage	ASP versus PWUS-P	1.61 (1.05–2.45)
	PWUS-C versus PWUS-P	0.93 (0.67–1.29)
Ensuring your data is kept private	ASP versus PWUS-C	1.48 (0.99–2.21)
	ASP versus PWUS-P	1.50 (1.04–2.16)
	PWUS-C versus PWUS-P	1.01 (0.75–1.36)
Transparency about when and how your data is collected, used, and disclosed	ASP versus PWUS-C	1.13 (0.76–1.68)
	ASP versus PWUS-P	1.54 (1.08–2.18)
	PWUS-C versus PWUS-P	1.36 (1.01–1.83)
Ensuring your anonymity	ASP versus PWUS-C	1.31 (0.88–1.93)
	ASP versus PWUS-P	1.56 (1.10–2.22)
	PWUS-C versus PWUS-P	1.19 (0.89–1.60)
Ability of apps like BRAVE to release your information such as your address only after you have a drug poisoning/overdose	ASP versus PWUS-C	1.67 (1.14–2.45)
	ASP versus PWUS-P	1.57 (1.11–2.22)
	PWUS-C versus PWUS-P	0.94 (0.71–1.25)
Connection to people of lived experience/peers	ASP versus PWUS-C	1.30 (0.88–1.90)
	ASP versus PWUS-P	1.55 (1.10–2.19)
	PWUS-C versus PWUS-P	1.20 (0.90–1.60)
Data privacy especially address and phone number	ASP versus PWUS-C	1.14 (0.79–1.66)
	ASP versus PWUS-P	1.13 (0.81–1.57)
	PWUS-C versus PWUS-P	0.98 (0.74–1.31)
Support service staffed by people with lived experience	ASP versus PWUS-C	0.68 (0.47–0.98)
	ASP versus PWUS-P	0.94 (0.68–1.31)
	PWUS-C versus PWUS-P	1.40 (1.05–1.86)
Data privacy and philosophies of care		
Access to mental health supports	ASP versus PWUS-C	1.54 (1.01–2.33)
	ASP versus PWUS-P	1.57 (1.08–2.29)
	PWUS-C versus PWUS-P	1.02 (0.75–1.39)
Ensuring EMS is sent instead of police in the event of a drug poisoning/overdose	ASP versus PWUS-C	1.21 (0.81–1.83)
	ASP versus PWUS-P	1.43 (0.99–2.06)
	PWUS-C versus PWUS-P	1.18 (0.87–1.60)
Access to treatment options	ASP versus PWUS-C	1.55 (1.02–2.34)
	ASP versus PWUS-P	1.60 (1.10–2.33)
	PWUS-C versus PWUS-P	1.04 (0.77–1.40)
Ability to get harm reduction supports wherever you may be at currently (home, work, shelter, etc.)	ASP versus PWUS-C	1.43 (0.94–2.16)
	ASP versus PWUS-P	1.98 (1.37–2.88)
	PWUS-C versus PWUS-P	1.39 (1.03–1.88)
Access to harm reduction supports including supplies, education, naloxone kits, and community resources	ASP versus PWUS-C	1.23 (0.82–1.84)
	ASP versus PWUS-P	1.62 (1.13–2.33)
	PWUS-C versus PWUS-P	1.32 (0.98–1.78)
Remaining arms-length from government agencies (e.g. police, health authorities)	ASP versus PWUS-C	0.91 (0.62–1.32)
	ASP versus PWUS-P	1.19 (0.85–1.66)
	PWUS-C versus PWUS-P	1.31 (0.98–1.75)
Substance usage		
Feeling safer while using substances	ASP versus PWUS-C	1.24 (0.84–1.82)
	ASP versus PWUS-P	1.37 (0.97–1.93)
	PWUS-C versus PWUS-P	1.10 (0.82–1.48)
Ability to use substances that some supervised consumption services can’t support (e.g. inhaled substances)	ASP versus PWUS-C	1.74 (1.15–2.61)
	ASP versus PWUS-P	2.30 (1.58–3.34)
	PWUS-C versus PWUS-P	1.33 (0.96–1.78)
Ability to use alone	ASP versus PWUS-C	0.99 (0.68–1.43)
	ASP versus PWUS-P	1.21 (0.87–1.69)
	PWUS-C versus PWUS-P	1.23 (0.92–1.64)
Ability to isolate and use substances in light of COVID-19	ASP versus PWUS-C	1.10 (0.76–1.60)
	ASP versus PWUS-P	1.59 (1.14–2.23)
	PWUS-C versus PWUS-P	1.45 (1.09–1.93)
Time to prepare substances for consumption while on the call for NORS	ASP versus PWUS-C	1.06 (0.73–1.54)
	ASP versus PWUS-P	1.34 (0.96–1.89)
	PWUS-C versus PWUS-P	1.27 (0.95–1.69)

### Accessibility and technical logistics/features

**Fig. 1:**
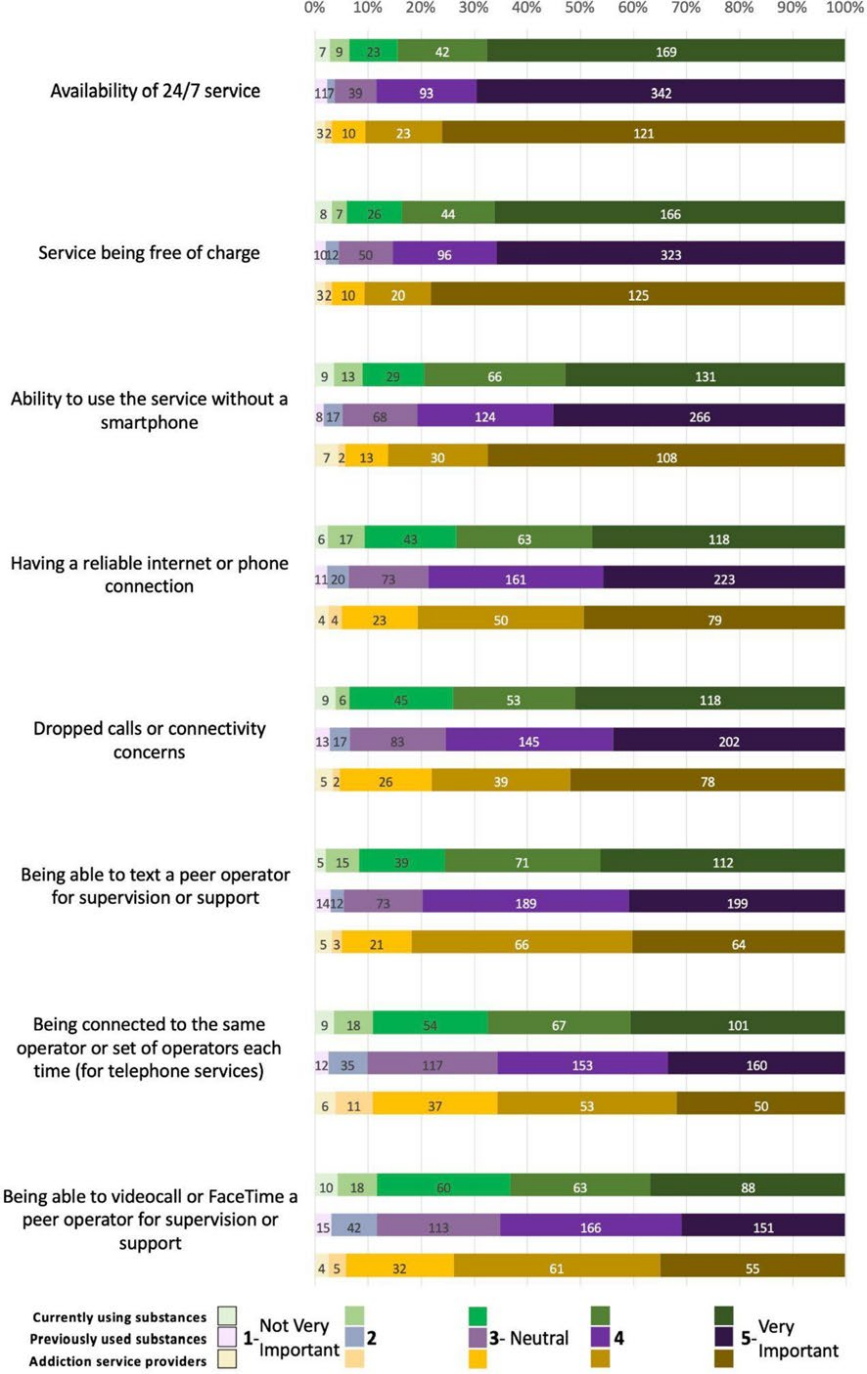
Accessibility and technological logistics

Most respondents from the three groups considered all accessibility and technical logistics elements important (Fig. 1). In order of importance, 24/7 service availability was the highest-ranked element across all groups, with 84% of PWUS-C, 88% of PWUS-P, and 90% of ASP ranking it as important. This was followed by the service being free of charge (83%, 85%, and 90%, respectively) and the ability to access the service without needing a smartphone (79%, 81%, and 86%, respectively). The next element was being able to text a peer operator (76%, 80%, and 82%, respectively), followed by the ability to have reliable internet access (73%, 77%, and 80%, respectively), concerns with dropped calls or connectivity issues (74%, 75%, and 78%, respectively), and being able to video call operators (64%, 65%, and 74%, respectively). Lastly, 67% of PWUS-C, 66% of PWUS-P, and 66% of ASP ranked being connected with the same operator across different use sessions as important.

ASP had significantly higher odds than PWUS-C of rating 24/7 service availability as an important service feature. ASP also had significantly higher odds of rating services being free of charge and the ability to use the service without a smartphone as important compared with both PWUS-C and PWUS-P. No significant differences were observed across groups for the importance of having a reliable internet connection, dropped calls or connectivity concerns, texting a peer operator, being connected to the same operator, or video calling.

### Overdose responses functionality

**Fig. 2:**
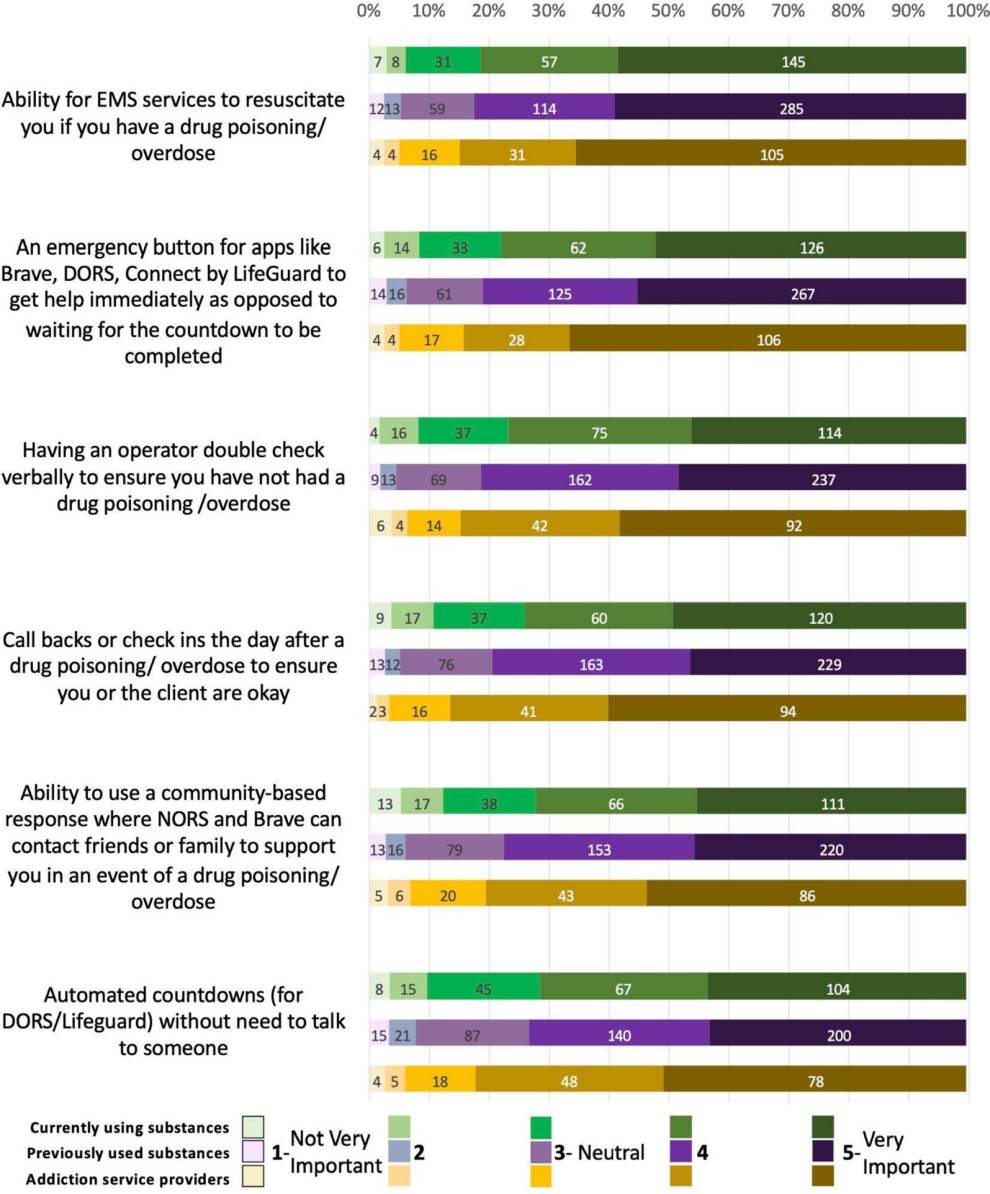
Overdose response functionality

Nearly all respondents across the interested parties ranked every overdose response element as important (Fig. 2). 81% of PWUS-C, 83% of PWUS-P, 85% ASP ranked the ability of EMS to successfully resuscitate individuals during an overdose as important, making it the highest-ranked element among all groups. This was followed by having emergency buttons to get immediate help without waiting for a complete countdown (78%, 81%, and 84%, respectively), having an operator verbally confirm with the client that no overdose has occurred (77%, 81%, and 84%, respectively), arranging callbacks or check-ins the day after an overdose (74%, 79%, and 87%, respectively), availability of community-based responses (72%, 78%, and 81%, respectively), and lastly, automated countdowns without needing to talk to someone (72%, 73%, and 82%, respectively).

ASP had significantly higher odds than both PWUS-C and PWUS-P of rating emergency buttons for immediate help, post-overdose check-ins, and automated countdowns as important service features. ASP also had significantly higher odds than PWUS-C of rating verbal double-checks by an operator to confirm no overdose has occurred and the ability to use a community-based response. There were no significant group differences in the importance of EMS’s ability to resuscitate during an overdose.

### Data privacy and philosophies of care

**Fig. 3:**
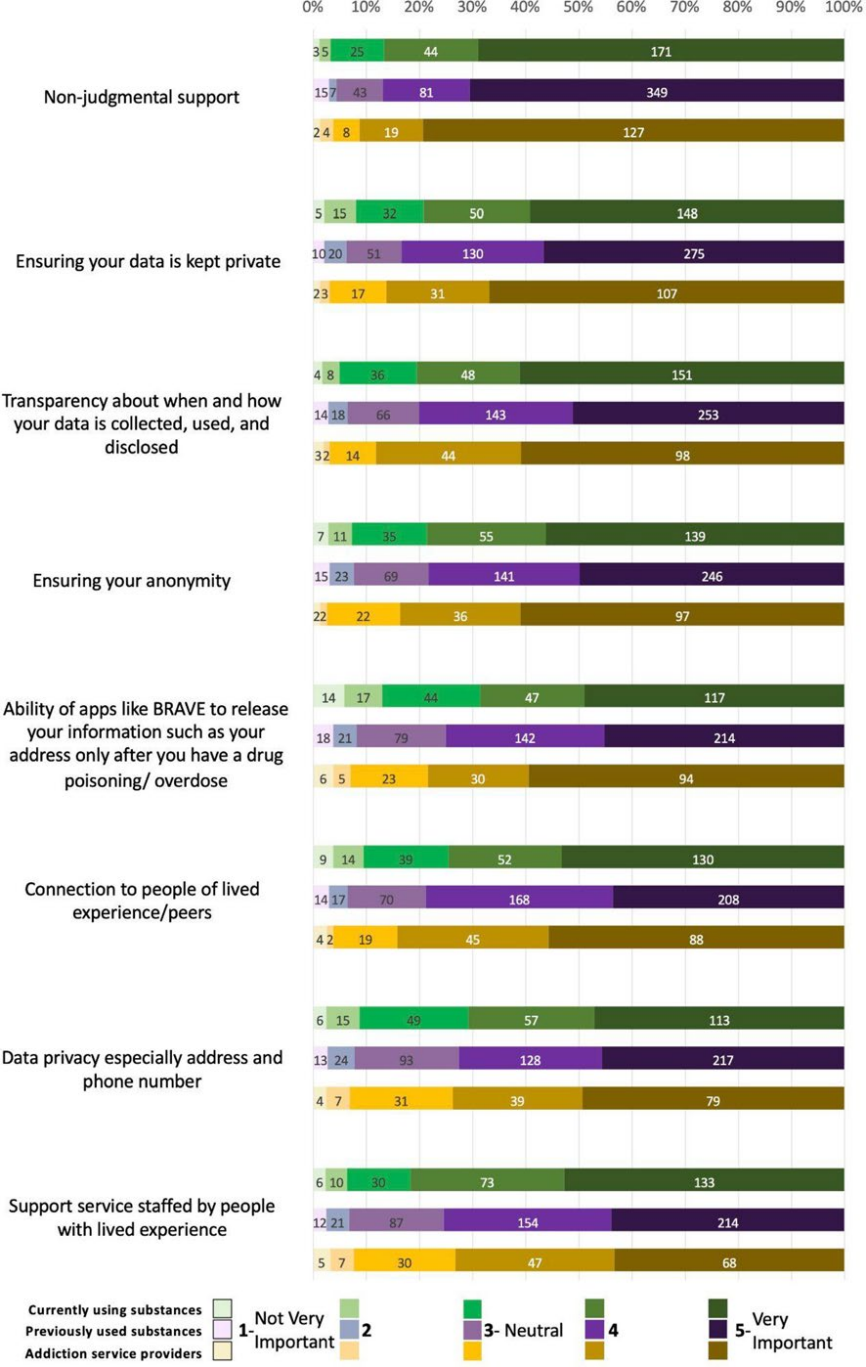
Data privacy and philosophies of care

A majority of respondents across all groups considered all elements in this category as important (Fig. 3). The highest-ranked element for all groups was non-judgmental support, with 87% of PWUS-C, 87% of PWUS-P, and 91% of ASP considering it important. This was followed by data privacy (79%, 83%, and 86%, respectively), data transparency (81%, 81%, and 88%, respectively), and anonymity (79%, 78%, and 84%, respectively). Additionally, 75% of PWUS-C, 79% of PWUS-P, and 84% of ASP ranked the connection to people of lived experiences as important, followed by having support staff/line operators with lived experience (82%, 75%, and 73%, respectively), the ability to release personal information only after an overdose (for response purposes) (69%, 75%, and 78%, respectively), and lastly, data privacy regarding phone number and address (71%, 73%, and 74%, respectively).

ASP had significantly higher odds than both PWUS-C and PWUS-P of rating non-judgmental support and the ability for apps to release information following an overdose as important service elements. ASP also had significantly higher odds than PWUS-C of valuing assurance of anonymity and connection to peers. Data transparency was rated as important by both ASP and PWUS-C, who had significantly higher odds of valuing this feature than PWUS-P. In contrast, PWUS-C had significantly higher odds than PWUS-P of rating services staffed by people with lived experience as an important element.

### Additional service supports

**Fig. 4:**
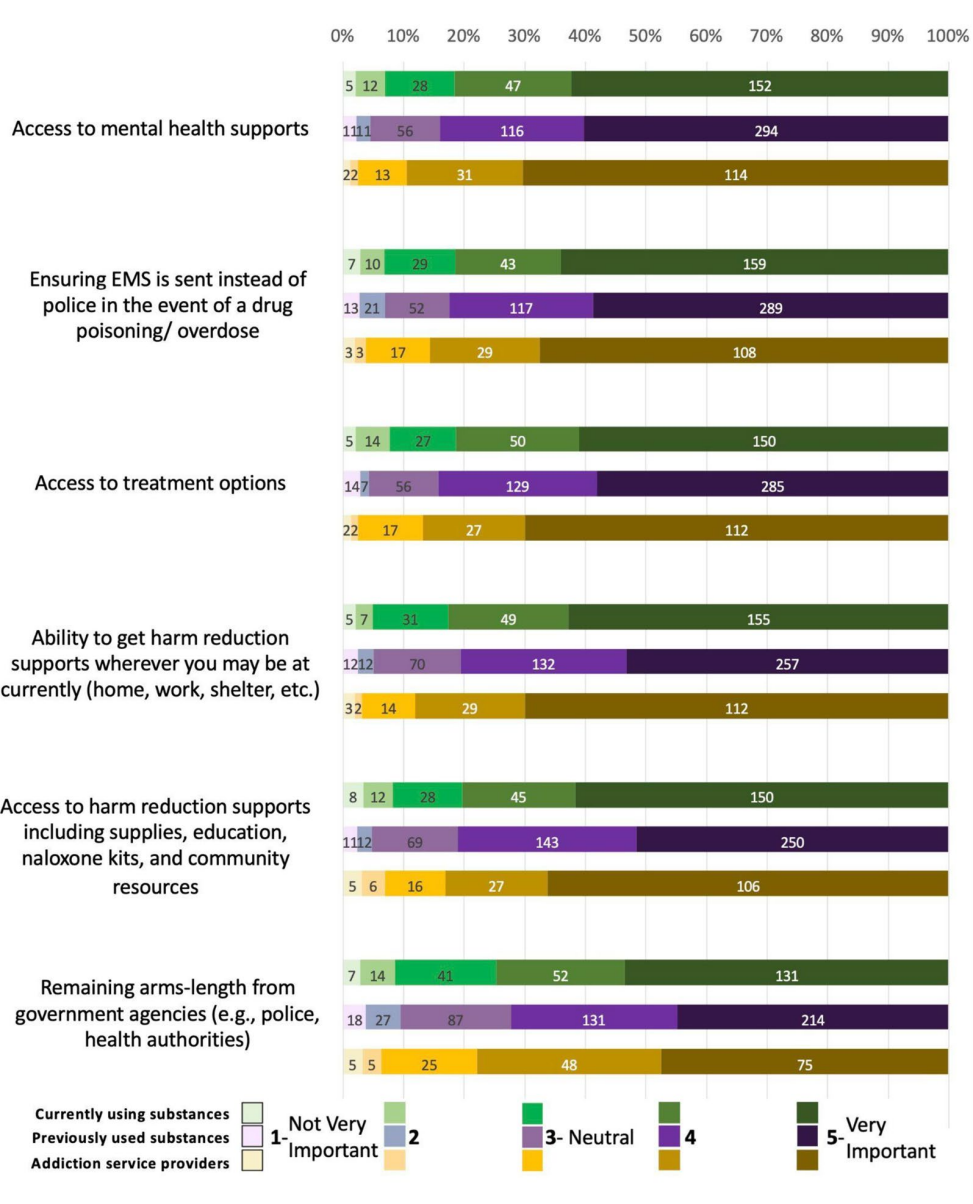
Additional service supports

Most respondents from all groups considered additional service support elements important (Fig. 4). Access to mental health support was ranked the highest element in this category, with 82% of PWUS-C, 84% of PWUS-P, and 90% of ASP ranking it as important. This was followed by access to treatment options (81%, 84%, and 87%, respectively) and ensuring EMS is sent instead of the police (82%, 83%, and 86%, respectively). Subsequently, 83% of PWUS-C, 81% of PWUS-P, and 87% of ASP ranked the ability to get connected to harm reduction supports near the client’s location as important, and this was followed by obtaining harm reduction support, such as supplies, education, or community resources (80%, 81%, and 83%, respectively), and lastly, maintaining distance from government authorities, such as police and healthcare bodies (75%, 72%, and 78%, respectively).

ASP had significantly higher odds of rating access to mental health supports and treatment options as significant service elements than PWUS-C and PWUS-P. ASP also had significantly higher odds of rating the ability to get harm reduction support and access to harm reduction supplies as a significant element than PWUS-P. There were no significant associations for ensuring EMS is sent over police and remaining arms-length from government agencies.

### Substance usage

**Fig. 5:**
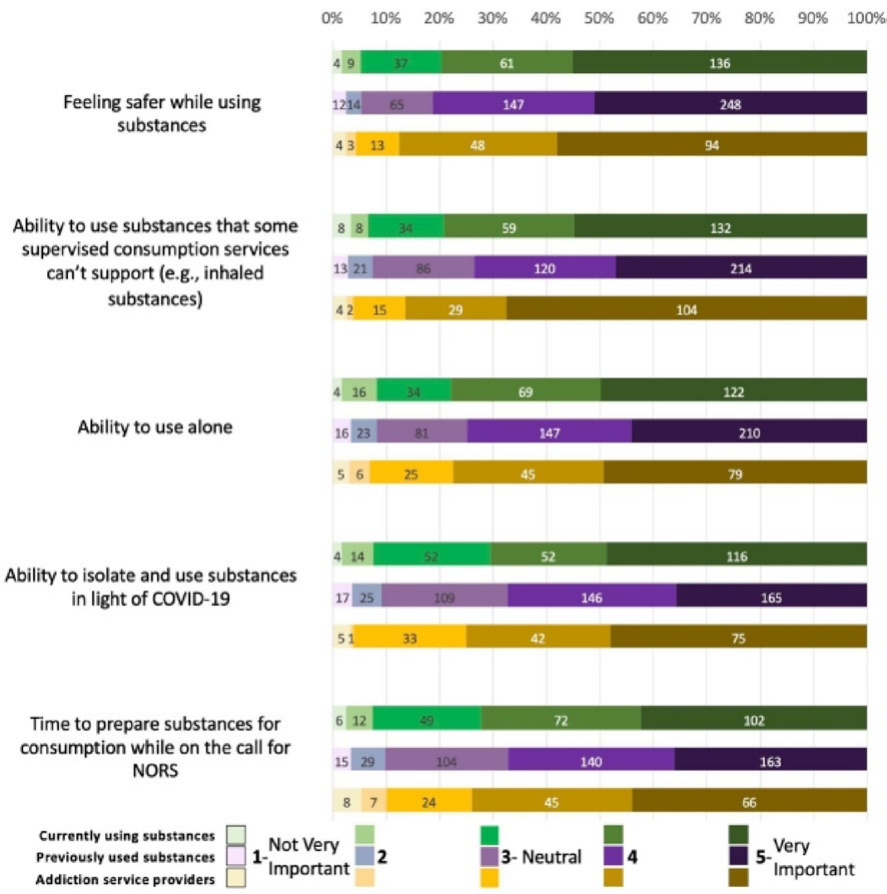
Substance usage

Overall, when asking questions specific to substance usage, most respondents from each group ranked all elements as important (Fig. 5). 80% of PWUS-C, 81% of PWUS-P, and 88% of ASP ranked feeling safer while using substances as important, making it the highest ranked element in this category. This was followed by the ability to use substances in routes that some SCS can't support (79%, 74%, and 86%, respectively), the ability to use substances alone (79%, 75%, and 78%, respectively), the ability to isolate and use substances in light of COVID-19 (71%, 67%, 75%, respectively), and lastly, time to prepare substances while on the call with NORS (72%, 61%, and 74%, respectively).

ASP had significantly higher odds of rating ability to use substances some SCS can’t support as a significant element than PWUS-C and PWUS-P. Additionally, PWUS-C and ASP had significantly higher odds of rating the ability to isolate and use substances in light of COVID-19 as a significant service element compared to PWUS-P. There were no significant associations between the time to prepare substances, feeling safer when using substances, and ability to use alone.

## Discussion

This study aimed to deepen the understanding of specific service elements of ORHAs by evaluating how PWUS-C, PWUS-P, and ASP view the importance of specific elements of ORHAs. The findings reveal that the interested groups studied were almost entirely in agreement about the importance of the examined potential service aspects identified during survey development, though the exact order of importance saw some minor variation. Results also indicated that ASP had higher odds of ranking certain elements as more important compared to other groups. Specifically, views on peer support, data privacy, EMS involvement, mental health support, and harm reduction service access provide key insights that can help shape ORHAs to meet the needs of all groups and prioritize the most impactful elements.

Peer support was an element ranked highly in importance across all groups and this finding is consistent with previous research on the value of connecting with individuals with lived experiences. Literature has shown that peer support workers can form meaningful relationships with PWUS, ones that are based on both trust and shared experiences [21]. Our findings are consistent with prior research showing that PWUS value the sense of common understanding from people of lived experience facilitated by peer support when using ORHAs, as this creates a space where they feel more connected and supported [9]. Additionally, shared lived experiences enhance feelings of safety and comfort, emphasizing the importance of this element in virtual harm reduction [22]. Peer support workers can also provide personalized care while bridging gaps between PWUS and other services [23]. Peer support being ranked highly in our results also aligns with previous literature exploring perspectives towards ORHAs, where the trusting and non-judgmental space provided by hotlines is appealing to PWUS (Marshall et al., 2023). While some overdose response hotlines emphasize connecting PWUS with peer operators with lived experiences, overdose response applications that do not have a live operator may struggle to deliver a similar experience. Based on previous research and the findings of this study, receiving support from those with lived experiences should remain a central focus in ORHAs, as it represents a crucial element of effective virtual harm reduction strategies.

A finding coherent with the existing literature was related to data privacy and anonymity. In particular, a high percentage of respondents among all interested parties ranked data privacy as important. Prior research on ORHAs supports this, indicating that while people are generally concerned about privacy, they understand that specific data, such as location, are necessary to ensure their safety during emergencies [8]. By ensuring data is kept private for individuals who seek virtual harm reduction, ORHAs can continue to provide a safe space for PWUS. Previous research has also shown that perceived lack of privacy and confidentiality can act as a barrier to PWUS accessing SCS [24]. By prioritizing data privacy for ORHAs, these barriers can be reduced, encouraging more individuals to seek the support they need and ultimately enhancing the effectiveness of virtual harm reduction services. While data privacy was largely deemed important, fewer participants were as concerned about sharing phone numbers and addresses. ORHAs would struggle to be as effective if clients were unwilling to share contact and location information, and it seems that the interested parties involved in this study understand that. Relatedly, video calling a peer operator was ranked as less important than other elements, such as being able to text a peer operator. This could be because there is an assumption that texting has more privacy than video calling, which ultimately leads to texting being considered a more important element, though other explanations (e.g. perception of convenience or reliability) are also possible. These findings suggest that to enhance virtual harm reduction platforms, expanding accessible text support should be prioritized over video calling. Anonymity, on the other hand, refers to not collecting any personally identifying information [25]. Interestingly, while data privacy was consistently valued, anonymity, although still ranked important, was less of a concern for respondents. Aligning with past literature on NORS, research has also shown that individuals engaging with virtual harm reduction services are less concerned about remaining anonymous than one might suppose [26]. By prioritizing data privacy and balancing the need for personal information in overdose responses with the need for anonymity, ORHAs can continue to offer practical, trusted harm reduction services while incorporating the perspectives of multiple groups.

Ensuring EMS is sent instead of the police during an overdose was deemed important by a majority of respondents across all groups, and our findings align with research on police involvement in overdose responses. Prior studies on ORHAs have shown that police presence is a major barrier to uptake, with PWUS reporting they are more willing to use these services when there is a guarantee that law enforcement will not attend (Marshall et al., 2023). This is consistent with broader literature demonstrating that interactions between PWUS and the police have gotten better in some jurisdictions [27], hesitation to contact 911 during an overdose commonly remains, often due to fear of potential legal consequences should police accompany EMS [28, 29]. Research has found that PWUS who present at an overdose site fear they may be wrongly suspected of manslaughter or other criminal charges [30]. Moreover, literature also highlighted that PWUS report hesitance to seek help during an overdose due to fears of losing their children to child protective services [31]. Furthermore, PWUS have expressed a desire for compassionate and non-judgmental treatment from EMS during overdose responses, and for the absence of law enforcement [32]. Previous research indicates that healthcare responders, including paramedics, comprehend the complexity of substance use and emphasize the importance of listening to the lived experiences of those who use substances [33]. ORHAs can improve their ability to ensure comfort and safety of PWUS by creating a system where EMS is sent instead of the police, ultimately, enhancing and encouraging access to virtual harm reduction resources. For example, maintaining NORS safety-plan options that allow callers to choose between a community response (family or friends) or EMS can address immediate medical needs while reducing fears associated with law-enforcement involvement. Considering the promise and impact of overdose technologies reducing mortality, this approach can address the immediate medical needs during an overdose but also mitigate the fears associated with legal authorities, fostering a more supportive environment for individuals seeking help through ORHAs [34, 35].

Mental health services were also ranked important by respondents across all interested parties. This finding aligns with existing literature highlighting the importance of connecting PWUS with health and social service supports as part of effective harm reduction initiatives [8]. Mental health support has been emphasized as critical in harm reduction settings, as concurrent mental illnesses are significantly associated with substance use [36]. Furthermore, NORS has been shown to receive a substantial portion of calls that are already mental health-oriented, in addition to overdose supervision [37]. Therefore, building mental-health capacity into ORHAs is not just helpful, it’s necessary. Our findings show that, in addition to reducing mortality rates and enhancing overdose responses, connecting individuals to social services and addressing their comprehensive needs will enhance the value and impact of ORHAs. This comprehensive approach can address health concerns while also fostering long-term well-being for individuals affected by substance use and mental health challenges. Regarding harm reduction support, PWUS-C and PWUS-P viewed access to harm reduction as important. However, ASP placed greater importance on receiving this support than the other groups. This point is further emphasized as ASP ranked the ability to use substances unsupported by SCS as more important than other stakeholder groups did. Previous literature has shown that professionals in this field adopt evidence-based interventions, which include harm-reduction strategies [38]. This could explain the percentage of ASP ranking harm reduction-related ORHA elements as important, as it reflects values from their professional training. In addition to prioritizing mental health referrals, expanding harm reduction education within ORHAs may also be beneficial. Across multiple domains, ASP consistently placed greater importance on a wider range of ORHA service elements compared to PWUS-C and PWUS-P. This pattern may reflect differences in professional responsibility, risk management, and system-level perspectives between providers and individuals with lived experience.

## Limitations and future research

Although the large sample size was a strength, partial purposive sampling could have limited the external validity [39]. Given the large sample size, the magnitude of differences between groups may not be large enough for clinical significance, despite certain differences in rankings between groups being statistically significant. Additionally, we recognize that certain participants fit multiple group criteria (e.g. have lived experience and work in the field of addictions), and only being able to select one group may have been a limitation. Another limitation lies in the gender representation of ASP, as the study included a larger number of women. Previous literature has explored the perspectives of women and gender-diverse individuals who use substances on virtual harm reduction technologies, where the findings indicated that this demographic generally viewed ORHAs positively [40]. Future research could see if the gender of ASP plays a role in the difference of perspectives of harm reduction platforms, particularly between women in comparison to other genders. Furthermore, future research could focus on differences between regions and communities, as that comparison was not made in this study due to an unbalanced sample size. It was not our intention to differentiate sentiments in different regions, but rather to identify universal elements that may be important to ORHAs. In addition, future studies should explore the perspectives of a more diverse range of groups, including parents of adolescents who may utilize virtual platforms. Finally, and perhaps most importantly, future work should be undertaken to consolidate a set of planning, implementation, and operational standards that will best situate ORHAs to serve clients.

## Conclusion

This study highlights the importance multiple respondents place on various elements of ORHAs, reflecting the need to consider the studied elements when conducting planning, implementation, evaluation, and quality improvement work related to ORHAs. Our findings demonstrate collective support for the core elements of these technologies across groups. The results of this study provide an opportunity for improvements to virtual platforms, helping to make these novel technologies more responsive, accessible, and trusted. The findings are in line with qualitative studies that have identified a number of the examined service elements as important [8]. These insights can also serve as a foundation for standardization (but by no means a comprehensive framework) for virtual platforms, ensuring they prioritize the most important elements. By studying and integrating multiple perspectives, elements of ORHAs can be made to better support individuals who use virtual overdose prevention technologies.

## Data Availability

No datasets were generated or analysed during the current study.
